# Supplementary material for: Dielectrophoretic and Electrical Impedance Differentiation of Cancerous Cells Based on Biophysical Phenotype
Source: Biosensors (Basel). 2021 Oct 17;11(10):401. doi: 10.3390/bios11100401 (PMC8533712; doi:10.3390/bios11100401)
Supplement: Supplementary file 1 [file biosensors-11-00401-s001.zip › biosensors-1385263-supplementary.pdf]

biosensors

Supplementary Material

Dielectrophoretic and Electrical Impedance Differentiation of Cancerous Cells Based on Biophysical Phenotype

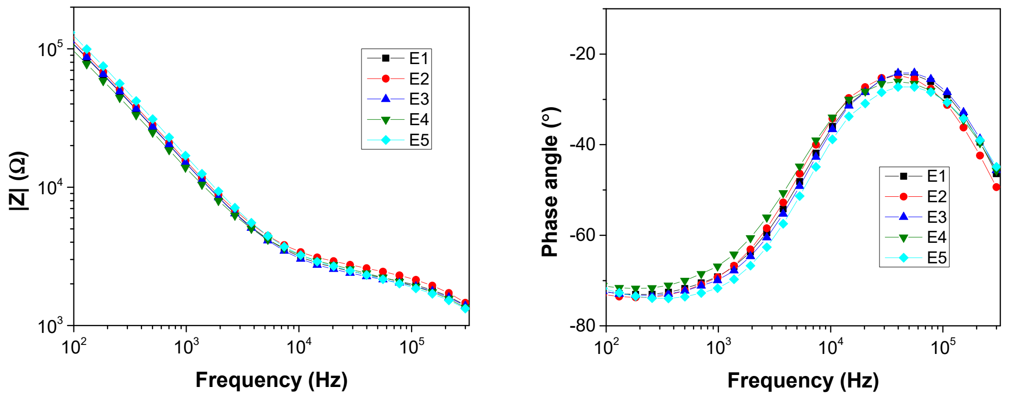

Figure S1. Microelectrodes reproducibility: Impedance magnitudes and phase angles of trapped T2 cancer cells at 9 Vpp, 1MHz obtained for 5 independent interdigitated microelectrodes.

Table S1. Summary of reproducibility tests by EIS technique performed at the level of 5 independent interdigitated microelectrodes.

	10^3 (Hz)			10^4 (Hz)			10^5 (Hz)		
	**Mean value**	**SD**	**RSD (%)**	**Mean value**	**SD**	**RSD (%)**	**Mean value**	**SD**	**RSD (%)**
**|Z| (Ω)**	15278.6	983.7	6.44	3196.5	121.3	3.79	1954.9	96.8	4.95
**Phase angle (°)**	-69.4	1.5	2.24	-35.9	1.7	4.88	-30.0	1.1	3.62

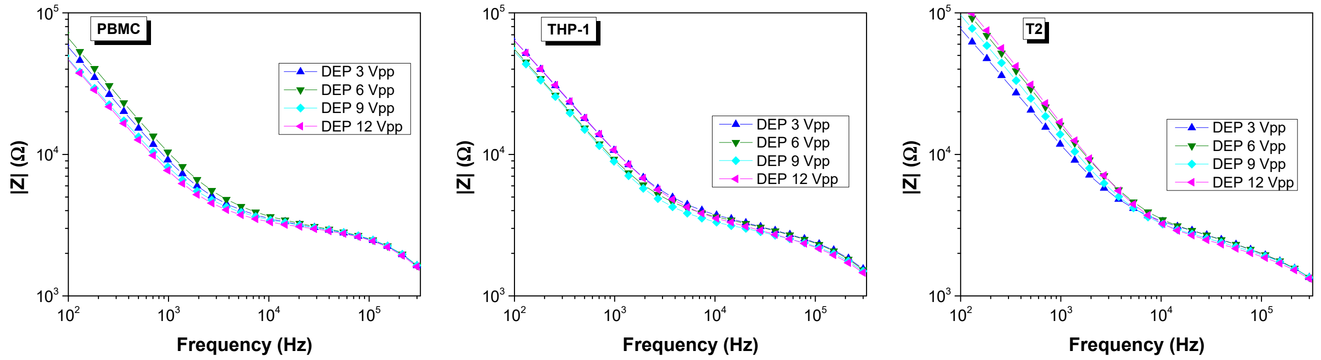

Figure S2. Impedance magnitudes of trapped PBMC, THP-1 and T2 cells at 3, 6, 9 and 12 Vpp.

Table S2. The reciprocal cell dielectric phenotype of cancer and normal blood cells.

**Cell type**	$1/R\phi$ **(10^6 m^{-1})**
PBMC	0.326 ± 0.016
THP-1	0.176 ± 0.007
Jurkat	0.097 ± 0.005
SW-403	0.087 ± 0.004
T1	0.089 ± 0.003
T1	0.096 ± 0.004
